# ANDA-Evaluating Facilitated Feedback Enhancement - a Cluster randomised Trial (ANDA-EFFECT): protocol for a cluster randomised trial of audit feedback augmented with education and support, compared to feedback alone, on acceptability, utility and health outcomes in diabetes centres in Australia

**DOI:** 10.1186/s13063-022-06910-9

**Published:** 2022-12-05

**Authors:** Matthew Quigley, Arul Earnest, Sofianos Andrikopoulos, Natalie Wischer, Sally Green, Sophia Zoungas

**Affiliations:** 1 School of Public Health and Preventive Medicine, Monash University, Melbourne, VIC 3004 Australia; 2 Australian Diabetes Society, Sydney, NSW 2000 Australia; 3 National Association of Diabetes Centres, Sydney, NSW 2000 Australia; 4 Diabetes and Vascular Medicine Unit, Monash Health, Clayton, VIC 3168 Australia

**Keywords:** Diabetes mellitus, Audit and feedback, Cluster randomised trial, Educational, Peer support, Feedback intervention

## Abstract

**Background:**

People living with diabetes must manage a range of factors for optimal control of glycaemia and to minimise the risk of diabetes-related complications. Diabetes practitioners are expected to follow guidelines for the key process of care and clinical outcomes, to help people living with diabetes achieve clinical targets. In Australia, the performance of diabetes centres against guidelines is evaluated by the Australian National Diabetes Audit, an annual clinical audit and feedback activity. Previous work has identified areas for improvement in the feedback provided to participating diabetes centres and suggested additional educational and support resources to assist in using audit feedback for the development of quality improvement activities. This cluster randomised trial will test the acceptability, utility and impact on selected clinical outcomes of the developed study intervention (audit feedback and a tailored educational and peer support cointervention).

**Methods:**

Two-armed cluster randomised trial with Australian Diabetes Centres that participated in the Australian National Diabetes Audit in 2021 as the clusters, stratified by location and type of centre. We aim to recruit 35 diabetes centres in each arm. Both the intervention and control arms will receive an augmented feedback report, accompanied by a partially pre-populated slide deck. In addition, the intervention arm will receive a tailored theory-based intervention designed to address identified, modifiable barriers to utilising and implementing the recommendations from diabetes audit feedback. The co-primary outcomes are (1) HbA1c at the patient level, measured at 6 months after delivery of the intervention, and (2) the acceptability and utility of the augmented feedback and cointerventions at the practitioner level, measured at 3 months after delivery of the intervention.

**Discussion:**

This trial aims to test the effects of systematic development and implementation of theory and evidence-informed changes to the audit feedback delivered to diabetes centres participating in an established national clinical diabetes audit. Potential benefits of improved audit feedback include more optimal engagement with the feedback by end clinical users which, ultimately, may lead to improvements in care for people living with diabetes.

**Trial registration:**

Australian and New Zealand Clinical Trials Registry ACTRN12621000765820. Prospectively registered on June 21, 2021

**Supplementary Information:**

The online version contains supplementary material available at 10.1186/s13063-022-06910-9.

## Administrative information

Note: the numbers in curly brackets in this protocol refer to SPIRIT checklist item numbers. The order of the items has been modified to group similar items (see http://www.equator-network.org/reporting-guidelines/spirit-2013-statement-defining-standard-protocol-items-for-clinical-trials/).

**Table:** 

Title {1}	**ANDA-Evaluating Facilitated Feedback Enhancement - a Cluster randomised Trial (ANDA-EFFECT): Protocol for a cluster randomised trial of audit feedback augmented with education and support, compared to feedback alone, on acceptability, utility and health outcomes in diabetes centres in Australia**
Trial registration {2a and 2b}.	This trial was prospectively registered with and the Australian and New Zealand Clinical Trials Registry (Trial Id: ACTRN12621000765820). Date of registration: June 21, 2021.
Protocol version {3}	Version 2.0, Date: May 14, 2021
Funding {4}	MQ is funded by an Australian Government Research Training Program (RTP) Domestic PhD Scholarship. The other authors report no specific funding with regards to this trial.
Author details {5a}	**Affiliations** 1. School Public Health and Preventive Medicine, Monash University, VIC, 3004, Australia 2. Diabetes and Vascular Medicine Unit, Monash Health, Clayton, VIC, 3168, Australia 3. Australian Diabetes Society, Sydney, NSW, 2000, Australia 4. National Association of Diabetes Centres, Sydney, NSW, 2000, Australia
Name and contact information for the trial sponsor {5b}	Sponsor: Monash University Contact information: Professor Sophia Zoungas, School of Public Health and Preventive Medicine, Monash University 553 St Kilda Road, Melbourne, VIC 3004, Australia Phone: +61 3 9903 0711 E-mail: Sophia.Zoungas@monash.edu
Role of sponsor {5c}	The sponsor and funder had no role in the study design, and will have no role in the collection, management, analysis, and interpretation of trial data. The writing of this protocol and the decision to submit the protocol for publication is also independent of the sponsor and funder, as will be any subsequent publications arising from this trial.

## Introduction

### Background and rationale {6a}

Diabetes is a common risk factor for cardiovascular disease, stroke, amputation and microvascular complications including retinopathy, neuropathy and nephropathy [1]. It is estimated that 68% of people over 65 years of age with diabetes will die from cardiovascular disease and 16% from stroke [1]. Optimal diabetes management requires people with diabetes to simultaneously control multiple factors including blood glucose, blood pressure, lipids and lifestyle factors such as diet and physical activity. Optimal control of these underlying risk factors is strongly associated with better outcomes and reduced cardiovascular disease, stroke and microvascular complications in people living with diabetes [1–5]. While people living with diabetes often self-manage many of the contributing factors, there is a high need for healthcare involvement to manage medications and monitor clinical markers; hence, the utilisation of healthcare services is higher among people with diabetes than in the general population [6]. Additionally, many people living with diabetes struggle to follow all recommended care processes, although following these processes is associated with lower rates of both inpatient and outpatient hospital presentations and improved microvascular and macrovascular outcomes [4, 5, 7, 8]. Interventions to support optimal management of risk factors in diabetes, both by people with diabetes and the healthcare services they access, therefore have the potential to improve health outcomes and reduce hospital presentations.

The Australian National Diabetes Audit (ANDA) is a longstanding clinical audit and feedback activity which has been administered by Monash University and the National Association of Diabetes Centres (NADC) since 2013. This clinical audit routinely collects de-identified information about clinical markers (e.g. HbA1c, blood pressure and lipid levels) and process of care measures (e.g. proportion of patients receiving annual retinal examination) among people living with diabetes in Australia who attend health services based in primary care, secondary or community care and tertiary referral centres. During a 4-week period in May through to July of each year, data is collected for all consecutive patients attending participating diabetes centres, using a standardised data collection form (Additional file 1). Data is collated and analysed, and participating centres then receive feedback in the form of an individualised benchmarking report which is made available to centres in an electronic format. Currently, the individualised report is approximately 200 pages long and provides detailed information on clinical performance in terms of clinical and process measures. Each measure is benchmarked to the mean achieved across all participating centres. Clinical performance indicators are presented in the form of tables and bar charts, allowing individual centres to interpret their data and undertake quality improvement activities as they see appropriate. While these individualised reports are only released to the individual diabetes centre, the de-identified data across all centres is also collated into a national report that is publicly available [9].

Audit and feedback activities such as ANDA are commonly employed in health care to inform quality improvement activities, based on the assumption that healthcare practitioners and services are motivated to engage in best practice but may not be aware of suboptimal performance [10]. In a 2012 Cochrane review of the effects of audit and feedback on professional practice and healthcare outcomes (140 studies), audit and feedback was reported to generate small but meaningful improvements in practice and patient health outcomes across a number of different conditions and settings [10]. For compliance with desired practice (dichotomous outcomes), there was a median 4.3% absolute increase in desired practice (IQR 0.5 to 16.0%). For compliance with desired practice (continuous outcomes), there was a median 1.3% absolute increase in desired practice (IQR 1.3 to 28.9%). For patient outcomes (continuous), the median percent change was 17% (IQR 1.5 to 17%) [10]. While these changes may seem small, at a population level, they represent a substantial healthcare gain. The review recommended future studies of audit and feedback compare different approaches to its delivery.

Benefits of a large-scale audit such as ANDA include the ability to show variation in practice and highlight areas where there may be a lack of concordance between evidence-based recommendations and actual care, resulting in suboptimal outcomes [11]. Previous analysis of the ANDA data has shown variation in practice and outcomes and evidence-practice gaps in the form of prescribing and treatment gaps for lipid-lowering medication and antihypertensives for people with type 2 diabetes [12]. In addition, ANDA has documented a high prevalence of various cardiovascular risk factors for people with type 1 diabetes participating in ANDA [13], along with suboptimal HbA1c for people with type 2 diabetes [12]. Descriptive data from pooled ANDA reports show suboptimal mean HbA1c, blood pressure and lipid levels, with little change over time [9, 14, 15]. This lack of change in key clinical measures including HbA1c over multiple cycles of ANDA audit and feedback suggests that the current form of feedback may not be fully translating into reduced practice variation and optimal health outcomes.

Given that clinical and process of care outcomes for many centres indicate room for improvement with little change over time resulting from ANDA, there is a need to explore alternative, potentially more effective methods of audit feedback delivery that meet the needs of diabetes centres and stimulate quality improvement (QI) activities in areas where practice is suboptimal. Our previous qualitative study [16] used an approach based on the Consolidated Framework for Implementation Research (CFIR) [17] and sought to identify (i) how diabetes centres use the current ANDA feedback to inform QI activities, (ii) the barriers and enablers to optimal use of diabetes audit feedback in these centres, (iii) the data needs of the stakeholders, (iv) what format might be more beneficial for stakeholders to interpret and act on the feedback provided and (v) whether there are other feedback or practice improvement interventions that may assist.

The main desired changes to feedback we identified were a shorter report and a more engaging, simplified data visualisation style. Barriers to the use of feedback included access and knowledge about how to use the data provided to inform the development of QI activities. Perceived enablers of implementation included leadership engagement, peer mentoring and support to share experiences of using ANDA data to inform QI activities, and external policy and incentives to encourage participation, such as recognition of ANDA participation by professional and accreditation bodies. Potential cointerventions to augment the feedback and support QI development included exemplars from clinical change champions and peer leaders and educational resources to help facilitate change [16]. These results support our contention that the existing ANDA audit feedback may not be used optimally and inform the redesign of ANDA feedback and the development of educational and community of practice resources based on the needs of diabetes centres, underpinned by the CFIR and contemporary audit and feedback literature.

### Objectives {7}

#### Hypothesis

The systematic, theoretically underpinned, development and delivery of a tailored intervention of enhanced audit feedback and an educational and community of practice cointervention for diabetes healthcare professionals will improve the acceptability and utility of ANDA audit feedback and will subsequently improve healthcare professional practice and patient outcomes when compared to receiving audit feedback alone.

#### Objectives

This cluster randomised trial will test the acceptability, utility and impact on selected clinical outcomes of the developed study intervention (audit feedback and a tailored educational and community of practice cointervention). The specific objectives of this project are:

1. To determine whether the implementation of redesigned ANDA feedback with or without the addition of tailored educational and community of practice resources leads to an improvement in HbA1c at 6 months and selected other clinical outcomes, when compared to previous audits
2. To determine whether the redesigned ANDA feedback and tailored educational and community of practice resources are associated with improved practitioner perceptions regarding the acceptability and utility of ANDA feedback

### Trial design {8}

As the trial interventions will target participants who receive and act on ANDA feedback (health service staff and practitioners), effectiveness is best evaluated through a cluster randomised trial with clustering at a centre level. The framework of this trial is a superiority trial with 1:1 allocation.

## Methods: participants, interventions and outcomes

### Study setting {9}

The setting for this trial is Australian diabetes centres participating in ANDA.

### Eligibility criteria {10}

#### Inclusion criteria

##### Diabetes centres

Australian diabetes centres (Centres of Excellence, primary, secondary and tertiary care services) registered with the National Association of Diabetes Centres (NADC) will be eligible for inclusion in the trial if the following criteria are met: (i) at least one representative of the practice (the designated contact person for ANDA) provides written informed consent; (ii) the practice participates in ANDA in 2021 (irrespective of whether they have or have not participated in previous years). Differences between types of eligible diabetes centres are shown in Table 1.

**Table 1:** Differences between types of eligible diabetes centres (based on the criteria set out by the NADC)

Type of centre	Definition
Primary care centres	General practices or community health centres that work closely with the local general practitioners to provide care for people with diabetes
Secondary care centres	Services with a range of full and/or part-time diabetes staff but who often do not have an endocrinologist as part of their usual team
Tertiary care centres	Diabetes centres that have the full range of diabetes service providers including endocrinologists, diabetes nurse educators, psychologists, dietitians and podiatrists on staff (full time) and have been accredited by the NADC
Centres of Excellence	Recognised diabetes centres that have demonstrated excellence in education, research, service delivery, practice/policy development and education. These centres must be tertiary-level facilities

##### Patient data

The study will not recruit patient participants. Rather, patient data for pre-determined fields relating to type 1 or type 2 diabetes mellitus care among people aged 18 years and over will be extracted from existing routinely collected and de-identified (coded) data reported as part of the ANDA annual audit.

#### Exclusion criteria

We are not collecting any additional data directly from patients, and we will not utilise ANDA data relating to people with gestational diabetes mellitus (GDM) or diabetes of unknown type. There are no additional exclusion criteria related to diabetes centres or to patient data.

### Recruitment {15}

Formal invitations will be sent out by the ANDA Secretariat to the site leads/representatives of eligible sites to provide an explanatory statement of the research, a Participant Information and Consent Form (PICF) detailing the exact nature of the study; what it will involve for the participating centre; the implications and constraints of the protocol; and any risks involved in taking part and contact details for the project lead.

### Who will take informed consent? {26a}

Participation in this research is completely voluntary and there are no consequences for those centres who choose not to participate. Sites who wish to participate in this research will notify the project lead of their intent to participate and consent through the return of a signed PICF via email to the ANDA Secretariat or the project lead. The project lead will countersign and return the PICF to participating centres. Details and documentation of participating sites will be maintained by the ANDA Secretariat to maintain the coded nature of enrolment (i.e. the project lead will know the names of individual staff participating, but not the unique ANDA identifying number for the centre they work for).

Sites will be asked to respond within 7 days, after which time a reminder email will be sent. Subsequent reminders will be sent up to the time of the end of the 6-week recruitment period.

### Additional consent provisions for collection and use of participant data and biological specimens {26b}

No biological specimens will be collected as part of this trial or sub-study. The additional consent provisions for collection and use of (non-patient) data in an associated sub-study to validate the survey instrument are detailed in the section ‘Reliability and validity of survey instrument (sub-study)’.

## Interventions

### Explanation for the choice of comparators {6b}

ANDA data has shown room for improvement in key processes and clinical outcomes over the previous 8 years; hence, there is an urgent need for reformatting the feedback. Our formative work with ANDA centres identified desired changes to data presentation in ANDA feedback. The comparator will hence be ANDA feedback only, but this feedback will be redesigned to meet these identified needs.

### Intervention description {11a}

#### Interventions

##### Feedback report

Both the intervention and control groups will receive the redesigned ANDA feedback report. This feedback report will be informed by the formative qualitative study [16] and developed by the lead author in consultation with the investigator group, including clinicians and an audit and feedback expert. The draft design of the report will then be modified based on their advice about clarity and feasibility, in a co-design process. The redesigned feedback will be configured for an automatic generation process by the ANDA data management team and will be delivered electronically to the contact person for each participating diabetes centre as a PDF document at one timepoint (December 2021).

##### PowerPoint slide deck

A partially pre-populated PowerPoint slide deck template to facilitate presentation of data within clinical practice teams will also be provided with the feedback report. The slide deck will be developed by the project lead in consultation with the ANDA-EFFECT investigator group and will allow diabetes centres to enter the data from their ANDA feedback report into the PowerPoint template.

#### Control arm

Standard treatment/care: Participating diabetes centres randomised to the control arm will only receive the feedback report and PowerPoint slide deck, as above.

#### Intervention arm

In addition to the feedback report and PowerPoint slide deck, participating diabetes centres randomised to the intervention arm will receive a tailored theory-based intervention designed to address identified, modifiable barriers to utilising and implementing the recommendations from ANDA feedback. The design of the intervention is underpinned by a formal qualitative study to elicit current quality improvement practices and barriers to implementation of feedback [16].

##### Intervention components

The intervention comprises a package of educational resources and community of practice forums, delivered on the NADC website. These resources will include:

- A 45-min QI webinar will be developed to guide participants, including short instructional videos to guide participants through understanding their data, along with presentations delivered by external QI experts
- Audio-visual stories from clinical change champions from a range of metropolitan and regional services in different states in Australia, identified through prior NADC involvement. These stories will describe how change champions have used ANDA data to facilitate QI activities
- Peer-led community of practice forums will be provided on the NADC website to facilitate participants to share knowledge and ideas related to the effective use of ANDA data. These forums will be moderated by the project lead

These resources will be produced by the project lead and the investigator group in collaboration with the NADC to maximise their clinical outreach. To prevent contamination between study groups, the intervention resources will be password-protected to only be available to intervention sites.

##### Delivery

**Fig. 1:**
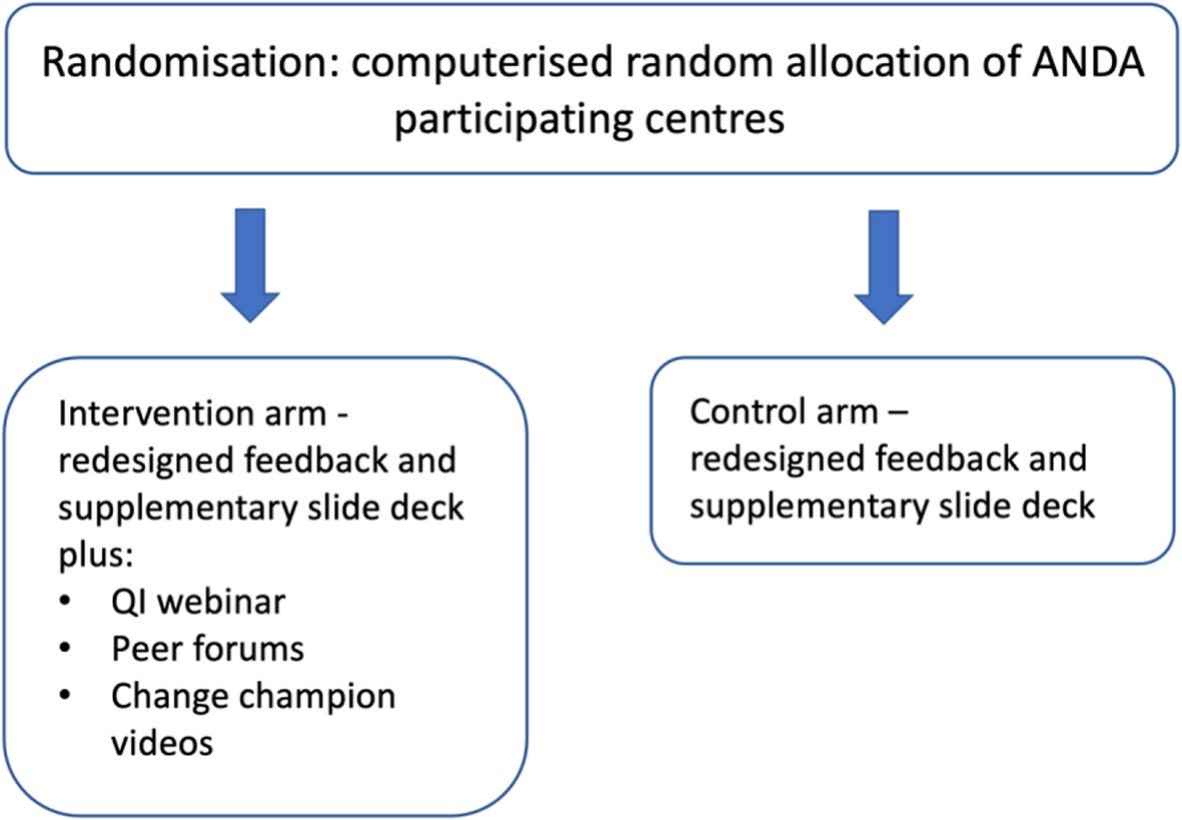
Overview of ANDA-EFFECT trial arms

The intervention resources will be made available to participants in the intervention arm by the ANDA research team approximately 3 months before they receive their redesigned ANDA audit feedback, to allow time for familiarisation with the resources. These resources will remain accessible to participants in the intervention arm following the delivery of ANDA audit feedback. There are no a priori plans for centre-specific tailoring or modification of the intervention resources. An overview of the trial arms is shown in Fig. 1.

#### Expectation of engagement

The expectation of participating centres will be made clear in the explanatory recruitment statement. Centres participating in the intervention arm will be expected to complete a minimum of three activities from the intervention resources, including:

- Attendance at the QI webinar—this will be hosted initially in the evening, followed by a live question and answer session. The webinar will be replayed with a live question and answer session during a lunch hour, to facilitate maximum uptake
- Viewing of at least one ‘clinical change champion’ video
- Participation in at least one peer-led forum

We anticipate that these activities will take up to 80 min in total, which could be comprised of multiple 5–10-min viewing blocks.

### Criteria for discontinuing or modifying allocated interventions {11b}

There are no criteria for discontinuing or modifying the allocated interventions, as the interventions are delivered to participating diabetes centres, not individual patients.

### Strategies to improve adherence to interventions {11c}

The trial will be promoted by the clinical partners (NADC) to their members who have participated in ANDA in 2021. A potential limitation of the recruitment strategy is that this is a pragmatic trial utilising an existing audit and feedback activity, where participation in ANDA 2021 has been impacted by the effects of COVID-19 on clinical practice [18]. As such, there is a lower than usual number of eligible diabetes centres from which to recruit, and the ANDA-EFFECT investigator group acknowledge that it may be difficult to reach optimal sample size; however, all efforts will be made to maximise participation.

#### Intervention fidelity

The interventions will be delivered in a standard, pre-recorded manner to all participants and as such are not susceptible to fidelity delivery variability in the way that educational interventions delivered as live sessions can be [19]. Our fidelity evaluation will therefore focus on whether participants engage with the interventions, as detailed in the section ‘Methods in analysis to handle protocol non-adherence and any statistical methods to handle missing data {20c}’.

### Relevant concomitant care permitted or prohibited during the trial {11d}

This is a cluster randomised trial targeting clinical practices, not patients. As such, there are no restrictions on concomitant care.

### Provisions for post-trial care {30}

Not applicable as this trial is testing interventions aimed at the cluster (practice) level rather than the patient level. As such, patient care is independent of the trial and will be administered as per normal processes by participating diabetes centres.

### Outcomes {12}, including plans for assessment and collection of outcomes {18a}

#### Primary outcomes

To address the aim of the trial to assess the impact of the interventions on clinical outcomes, we will collect data on HbA1c levels of patients attending participating centres for care. To address the aim of the trial regarding practitioner perceptions, we will collect data related to the acceptability and utility of the interventions.

##### HbA1c at 6 months

HbA1c is routinely collected for patients from participating diabetes centres as part of ANDA. Baseline de-identified (coded) mean HbA1c percentage will be extracted for each participating diabetes centre from routine ANDA data collection in 2021. Follow-up data will be collected as part of the ANDA 2022 audit (6 months after delivery of the 2021 ANDA site reports).

##### Acceptability and utility of the intervention at 3 months

To assess how well the interventions address the issues raised in our formative work [16] at a practitioner level, the acceptability and utility of the intervention will be assessed via a 15-min online survey, which we will design specifically for this purpose. This survey will adapt on a survey (ENACT) used by a group of audit and feedback researchers with permission from the authors (personal communication) to assess the acceptability of online audit and feedback interventions [20]. The ENACT survey will be adapted to allow inclusion of parts of an existing ANDA questionnaire. Surveys will be completed 3 months after the delivery of site reports (feedback). Participants will rate a variety of factors about the feedback using Likert scales and open-ended text comments. The developed, combined survey will be validated for test/retest reliability and face validity prior to its use in outcome assessment (see sub-study below). The survey will be delivered to participants as an email link sent to the registered contact persons for the participating sites and will be conducted through REDCap [21], a secure web application for online survey and database management hosted at Monash University. The survey will be delivered 3 months after the delivery of the ANDA feedback reports.

#### Secondary outcomes

##### Other clinical and process of care outcomes

Other de-identified (coded) baseline clinical and process of care outcomes will be extracted from routine ANDA data collection in 2021, using the ANDA data collection form. These will include mean systolic blood pressure (mm Hg) and associated prescribing rates of hypertensives as well as mean total cholesterol, low-density lipoprotein (LDL) and high-density lipoprotein (HDL) (mmol/L) and the associated prescribing rates of lipid-lowering medications. In the same manner as the primary clinical outcome, follow-up data will be collected as part of the ANDA 2022 audit (6 months after the delivery of the 2021 ANDA site reports). All clinical measures included in the standard ANDA data collection form are collected independently of this trial, either by the participating diabetes centres or by the independent pathology services undertaking clinical testing for the participating centres as part of their routine care. As these results are submitted to ANDA in a coded (de-identified) manner, managed by the ANDA data management team and reported in aggregate, the ANDA-EFFECT investigator group do not have access to individual patient data for these variables and will extract mean clinical outcomes from the existing ANDA data set.

##### Exploratory outcomes

HbA1c at 18 months after delivery of intervention may be collected from the ANDA 2023 audit.

### Participant timeline {13}

**Fig. 2:**
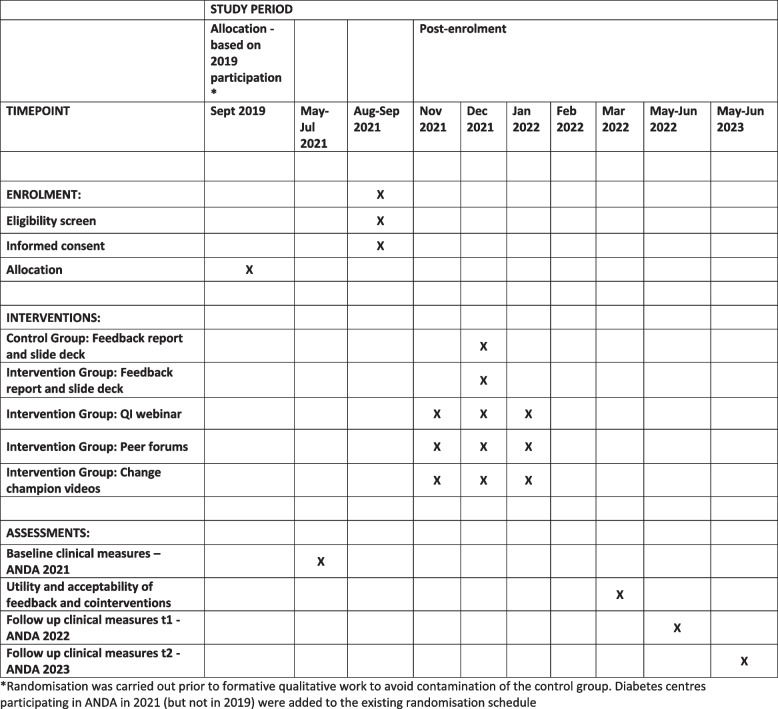
ANDA-EFFECT SPIRIT figure [22]

The participant timeline is presented in Fig. 2.

### Sample size {14}

Sample size calculation is based on a cluster randomised trial design with the difference in HbA1c as the primary endpoint. Assuming a mean difference in the intervention group of 0.5%, a total sample size of 3500 patients, 35 sites in each arm will provide at least 80% power assuming that the standard deviation of HbA1c is 2.3, the intracluster correlation coefficient is 0.070 and the coefficient of variation of cluster sizes is 0.730. We thus plan to recruit 78 diabetes centres: 39 in each arm to allow for a 10% non-response rate. Sample size calculation was based on the difference in means calculation with the level of significance set at 0.05. PASS V14 software [23] was used for the sample size calculation.

## Assignment of interventions: allocation

### Sequence generation {16a} and implementation {16c}

The random allocation sequence was generated by a senior biostatistician independent to the study in order to allocate the participating diabetes centres (clusters) into the experimental group (enhanced feedback and cointervention) and the control group (enhanced feedback) using random block sizes 2 and 4, stratified by type of centre (primary/secondary care vs tertiary care or Centres of Excellence (CoE)) and location (metropolitan or rural), based on centres who participated in ANDA 2019. Due to the low numbers of Centres of Excellences and the similarity to tertiary care centres, these types of centres will be combined for the purpose of stratification and analysis. We based the randomisation on 2019 ANDA participation and randomised prior to the formative qualitative work [16] to avoid potential contamination of the trial control group. As such, randomisation occurred prior to commencement. In addition, any centres who participated in ANDA for the first time in 2021 were allocated to either the intervention or the control arm as per the outlined randomisation method. Participants will be enrolled by the project lead as per section {26a}.

### Concealment mechanism {16b}

The independent statistician was provided with a file containing only the diabetes centre identification codes and stratification variables and no identifying information.

## Assignment of interventions: blinding

### Who will be blinded {17a}

The project lead and primary investigator will not be blinded to group allocation as they will be delivering the interventions, but they will only be aware of the site practice and outcome data pertaining to any given diabetes centre in a coded manner, as each participating diabetes centre has a unique identifying code. The ANDA Secretariat is the only person who has access to the codes linking diabetes centre IDs with their contact details and is not involved in the delivery of the intervention, assessment of outcome or analysis.

Other investigators and the biostatistician supervising the analysis will be blinded to group allocation. The data analysis team will be blinded to allocation details for the purpose of data analysis.

Participating diabetes centres will be blind to group allocation until the delivery of the interventions. Due to the nature of the intervention and control in this study, it will not be possible to blind the participating sites to the intervention they receive.

### Procedure for unblinding if needed {17b}

As this is a cluster randomised trial, sites (the participating diabetes centres) are not blinded due to the nature of the intervention.

## Data management

### Plans to promote participant retention and complete follow-up {18b}

Participating sites have the right to withdraw from the study at any time, with no consequences for withdrawal. Sites who do not participate or who withdraw from this trial (but not from ANDA) will receive the augmented ANDA feedback reports and full data appendix, as per standard processes. We have allowed for site withdrawals in the sample size calculations. If any site decides not to subsequently receive the intervention and withdraws, the de-identified (coded) clinical outcome data from the patients of this site will still be collected for this trial as part of the routine ANDA data collection that the sites have agreed to participate in.

### Data management {19} and confidentiality {27}

All data will be downloaded and electronically stored in line with applicable privacy principles. As part of ANDA data management, data collected with the standardised data collection form is checked by the data management team, with queries sent to participating diabetes centres regarding any data anomalies (such as out-of-range data). Once the data queries have been resolved, the ANDA dataset is checked by an independent data specialist [13].

All data will be password protected and will be housed on a secure Monash University Server, as per standard ANDA protocol [8]. The chief investigator, data manager and project lead will be the only researchers to have access to the data. All researchers on this project are involved in projects using research databases which have high levels of security. This electronic database will remain in password-secured storage for 7 years and then deleted from all backups.

### Plans for collection, laboratory evaluation and storage of biological specimens for genetic or molecular analysis in this trial/future use {33}

No biological specimens are being collected as part of this trial.

## Statistical methods

### Statistical methods for primary and secondary outcomes {20a}

#### Primary outcomes

Differences between the experimental and control groups will be analysed by the intention-to-treat approach. For the first co-primary outcome of difference in HbA1c, we will use the linear mixed effects model to compare the between-group difference in mean HbA1c at 6 months after delivery of the intervention while adjusting for clustering of patients with site by including a centre random intercept. Appropriate transformations of the data will be undertaken in the event of departure from normality. Only if the first co-primary outcome is statistically significant at a two-sided level of significance of <0.05, we will test the second co-primary of acceptability and utility of interventions for superiority using the chi-squared or Fisher’s exact test as appropriate. This will preserve the overall type 1 error at <0.05.

#### Secondary outcomes

Other secondary clinical endpoints (e.g. mean LDL-Ch) and exploratory outcomes (e.g. mean HbA1c at 18 months) will similarly be analysed using the linear mixed effects model.

### Interim analyses {21b}

No interim analyses are planned in this trial.

### Methods for additional analyses (e.g. subgroup analyses) {20b}

Additional subgroup analyses are not planned in this trial.

### Methods in analysis to handle protocol non-adherence and any statistical methods to handle missing data {20c}

The primary analysis is per intention to treat, i.e. any sites that are non-adherent to the intervention will be analysed accordingly to the intervention arm assigned during randomisation. Data analysis will be based on complete cases and there will be no imputation for missing data.

In order to understand the engagement with the interventions, a per-protocol sensitivity analysis will also be completed to assess the fidelity adherence to the intervention and components therein. This will be evaluated in two ways. Firstly, we will include self-report items in the acceptability and utility survey related to the use of each component of the intervention (e.g. Did you access the webinar?). This data will be triangulated with web analytics from the NADC website to show the proportion of participants who access each component of the cointerventions and the time that participants report spending on these components.

### Plans to give access to the full protocol, participant-level data and statistical code {31c}

In the interests of transparent reporting and reproducible research, the authors seek to make the full protocol publicly available through publication. The participant-level dataset will not be publicly available, due to the risk of inadvertently identifying participating centres (for example, where there may be limited numbers of participating diabetes centres within a given geographical jurisdiction). Reasonable requests for other data or code will be considered by the primary investigator, as per the ANDA Data Sharing Policy.

## Oversight and monitoring

### Composition of the coordinating centre and trial steering committee {5d}

“ANDA- Evaluating Facilitated Feedback Enhancement - a Cluster randomised Trial (ANDA-EFFECT): A cluster randomised trial of audit feedback augmented with education and support, compared to feedback alone, on acceptability, utility and health outcomes in diabetes centres in Australia” will be overseen by the ANDA-EFFECT investigator group and the ANDA Scientific Advisory Committee (ANDA-SAC).

The day-to-day management of this trial will be managed by the project lead and the ANDA-EFFECT investigator group, with assistance from the ANDA Secretariat and the ANDA data manager. Reports will be provided to the ANDA Operational Committee at fortnightly meetings.

The primary investigator will provide updates to the ANDA-SAC at each quarterly meeting and any publication arising from the research will be provided to the ANDA-SAC for endorsement prior to submission.

### Composition of the data monitoring committee, its role and reporting structure {21a}

A data monitoring committee was not deemed necessary in this trial, as this role is fulfilled by the ANDA Operational Committee.

### Adverse event reporting and harms {22}

This trial has been approved as a low-risk research endeavour. We do not anticipate any harms to participating clinical staff. Unlike a drug trial, this trial is utilising an existing audit and feedback activity and focuses on changes in data presentation and education to participating centres and their health practitioners. As such, the interventions do not directly target patients and we do not anticipate any harm to patients from this trial. Patient care will not be affected if a participating centre decides to withdraw from the trial, as the diabetes centres function independently from the trial. The Participant Information and Consent Form (PICF) provides contact details for the responsible Human Research Ethics Committee (HREC), Monash Health HREC, for participants who wish to report issues with the trial conduct.

### Frequency and plans for auditing trial conduct {23}

The project lead will complete annual progress reports for the responsible HREC (Monash Health HREC), who also have the capacity for an independent audit of the trial.

### Plans for communicating important protocol amendments to relevant parties (e.g. trial participants, ethical committees) {25}

Any amendments to the protocol will be submitted to the responsible Human Research Ethics Committee (Monash Health HREC) for approval, and the Australian and New Zealand Clinical Trials Registry (ANZCTR) entry will be accordingly updated.

### Dissemination plans {31a}

It is anticipated that the results of this trial will be published in peer-reviewed journals and/or presented in a variety of forums (such as conference presentations). The results of the trial will be published regardless of the outcome. In any publication and/or presentation, collated, de-identified findings of this trial will be presented. No information that could identify a specific person or diabetes centre will be included in publications or presentations. Copies of any manuscripts resulting from this work will be sent to participating diabetes centres. The embedding of this trial into usual ANDA practices and governance structure will facilitate translation of the findings into future ANDA activity.

## Reliability and validity of survey instrument (sub-study)

### Background

As a separate sub-study, we will test the reliability and validity of the developed acceptability and utility survey. As above, this survey will be based on a survey (ENACT survey) previously used by researchers to test the acceptability of online audit and feedback interventions [20].

The online survey will ask participants to rate a variety of factors about the site feedback provided as part of ANDA, using Likert scales and open-ended text comments. We will ask participants to rate the acceptability and utility of the feedback and cointerventions provided, both as an overall rating and with discrete section by section questions. Questions will address constructs such as whether the feedback meets user data needs, the clarity of data presentation, whether specific barriers have been addressed and whether users intend to use the feedback to inform QI interventions in clinical practice.

We plan to conduct this reliability and validity sub-study in a different population to that of the trial to reduce the burden on ANDA-EFFECT trial participants.

### Methods

#### Participants

Participants will be a similar professional group to those involved in ANDA-EFFECT, but do not need to be taking part in ANDA or ANDA-EFFECT to participate. We will recruit from the ANDA Scientific Advisory committee and selected hospital department heads.

#### Recruitment

Potential participants will be recruited via email, from a list of the ANDA Scientific Advisory committee and hospital department heads known to the investigators. This email will explain the purpose of the survey and the nature of the testing, including the requirements and burden of testing. This email will also contain a link to the survey, which will be conducted through REDCap [21], a secure web application for online survey and database management hosted at Monash University.

Reminder emails to complete the repeat survey for test/retest purpose will be sent on day 12 and day 14 after the initial email.

#### Burden of participation

Participants will be asked to complete the survey questions twice, within 15 days, to facilitate the test/retest data collection. This timeframe is believed to be long enough to prevent recall effects (i.e. the memory of the first test influencing the second test), but short enough to prevent a change in survey response due to changes in understanding of the basic constructs over time [24]. We envisage that the survey will take no longer to complete than 15 min. As such, the total burden to participants is approximately 30 min. No other activity will be required of participants.

#### Consent

Completion of the survey will constitute informed consent. All information about the purpose of the survey and use of data will be provided to participants as an introduction to the survey. Survey participants will not be identified, and potential participants will be free to choose to participate or not participate, with no adverse consequences for non-participation. Survey data will be used only for the purpose of reliability and validity assessment and responses will not be analysed with respect to reported practices or beliefs.

#### Analyses

Test/retest reliability will be evaluated through examination of the data from each participant across the 2 timepoints. To assess the reliability of the acceptability and utility survey, we will calculate Cohen’s kappa statistic for categorical scales and the intra-class correlation coefficient for continuous scales.

Face validity will be assessed by asking participants if selected questions within the acceptability and utility survey accurately represent the construct they are intended to represent. By using participants with similar content expertise to the participants in ANDA-EFFECT, we will be seeking opinions from suitably qualified experts. Internal consistency will be tested using Cronbach’s alpha to determine how closely correlated the sets of survey questions related to a given construct are.

#### Publication and dissemination

It is anticipated that the reliability and validity findings will be published in peer-reviewed journals and presented at conferences, but no identifiable data will be released.

## Discussion

The ANDA-EFFECT trial aims to test the effects of systematic development and implementation of theory and evidence-informed changes to the audit feedback delivered to diabetes centres participating in an established national clinical diabetes audit. This feedback will be directly influenced by our prior qualitative work which elucidated some of the barriers to the use of the audit feedback currently provided and contemporary audit and feedback literature. Potential benefits of improved audit feedback include more optimal engagement with the feedback by clinicians and diabetes centres which, ultimately, may lead to improvements in care for people living with diabetes.

## Trial status

Protocol version: 2.0. Recruitment commenced for this trial on August 9, 2021. We anticipate a 6-week recruitment period.

## Supplementary Information

**Additional file 1.** ANDA 2021 data collection form.

## Data Availability

The project lead and the primary investigator will have access to the final trial dataset.

## Abbreviations

ANDA-EFFECT: ANDA-Evaluating Facilitated Feedback Enhancement - a Cluster randomised Trial
RTP: Research Training Program
ANDA: Australian National Diabetes Audit
NADC: National Association of Diabetes Centres
HbA1c: Haemoglobin A1c
QI: Quality improvement
CFIR: Consolidated Framework for Implementation Research
GDM: Gestational diabetes mellitus
PICF: Participant Information and Consent Form
LDL: Low-density lipoprotein
HDL: High-density lipoprotein
COE: Centre of Excellence
LME: Linear mixed effects
ANDA-SAC: ANDA Scientific Advisory Committee
HREC: Human Research Ethics Committee
ANZCTR: Australian and New Zealand Clinical Trials Registry
