# Supplementary material for: ANDA-Evaluating Facilitated Feedback Enhancement - a Cluster randomised Trial (ANDA-EFFECT): protocol for a cluster randomised trial of audit feedback augmented with education and support, compared to feedback alone, on acceptability, utility and health outcomes in diabetes centres in Australia
Source: Trials. 2022 Dec 5;23:976. doi: 10.1186/s13063-022-06910-9 (PMC9720980; doi:10.1186/s13063-022-06910-9)
Supplement: Supplementary file 1 — Additional file 1. ANDA 2021 data collection form. [file 13063_2022_6910_MOESM1_ESM.pdf]

SAMPLE

17880

ANDA-AQCA 2021
Australian National Diabetes Audit - Australian Quality Clinical Audit

Section 1. Patient Demographics

Patient ID ☐☐☐☐☐☐☐☐☐☐☐☐☐☐☐☐ Site ID ☐☐☐ Staff Initials (optional) ☐☐☐☐

1.1 Date of birth ☐☐ / ☐☐ / ☐☐☐☐ (d d / m m / y y y y)

1.2 Sex ☐ Male ☐ Female *if FEMALE* → **1.2.1 Currently pregnant** ☐ No ☐ Yes

1.3 Date of visit ☐☐ / ☐☐ / 2 0 2 1 (d d / m m / y y y y)

1.4 Initial visit ☐ No ☐ Yes

1.5 Aboriginal/Torres Strait Islander ☐ No ☐ Yes

1.6 Country of birth ☐☐☐☐☐☐☐☐☐☐☐☐☐☐☐☐☐☐☐☐

1.7 NDSS member ☐ No ☐ Yes

1.8 DVA patient ☐ No ☐ Yes

Section 2. Diabetes Type & Management

2.1 Date of diagnosis ☐☐ / ☐☐☐☐ (m m / y y y y)

2.2 Type of diabetes ☐ Type 1 ☐ Type 2 ☐ GDM ☐ Don't know ☐ Other

2.3 Glucose monitoring *(Select all that apply)* ☐ None ☐ Finger pricking ☐ CGM ☐ Flash Glucose Monitoring

2.3.1 If using Flash/CGM, proportion of time using sensors ☐ <50% ☐ 50-75% ☐ >75-100%

2.4 Management method *(Select all that apply)*
☐ Diet only ☐ Sulphonylurea ☐ Insulin → **2.4.1 Insulin duration** ☐☐ years ☐☐ months
☐ Metformin ☐ SGLT2 inhibitor ☐ GLP1 agonist
☐ Acarbose ☐ Thiazolidinedione ☐ DPP4 inhibitor

2.4.2 Insulin mode *(Select all that apply)* ☐ Basal ☐ Basal bolus ☐ Hybrid closed loop system ☐ Pump ☐ Pre-mixed insulin

Section 3. Height, Weight & Smoking Status

3.1 Weight ☐☐☐ . ☐ kg

3.2 Height ☐ . ☐☐ m

3.3 Smoking status ☐ Current ☐ Past ☐ Never

If current OR past smoker

3.3.1 Number of years spent smoking ☐ <5 years ☐ 5-10 years ☐ 11-20 years ☐ >20 years

Section 5. Diabetic Eye Disease - last 12 months

	No	Yes
5.1 Attended optometrist/ophthalmologist	☐	☐
5.2 Fundus examination	☐	☐
5.3 Retinopathy	☐	☐
5.4 Treatment for retinopathy	☐	☐
5.5 Right or left cataract	☐	☐

Section 7. Medications & Lipids

	No	Yes	Contraindicated
7.1 Aspirin	☐	☐	☐
7.2 Other anti-platelets	☐	☐	☐
7.3 Anti-coagulants	☐	☐	☐
7.4 Lipid lowering Rx	☐	☐	
if YES → 7.4.1 Statin	☐	☐	☐
7.4.2 Fibrate	☐	☐	☐
7.4.3 Ezetimibe	☐	☐	☐
7.4.4 Fish oil	☐	☐	☐
7.5 Lipids measured	☐	☐	

if YES → **Complete below:** (Not available)

7.5.1 Total Cholesterol ☐☐ . ☐ mmol/L OR ☐ Not available

7.5.2 LDL ☐☐ . ☐☐ mmol/L OR ☐

7.5.3 HDL ☐ . ☐☐ mmol/L OR ☐

7.5.4 Triglycerides ☐☐ . ☐ mmol/L OR ☐

7.5.5 Were the above fasting lipids? ☐ No ☐ Yes

Section 4. Blood Pressure

4.1 Blood pressure (Most recent, measured after 5 mins sitting) ☐☐☐ / ☐☐☐ mmHg

4.2 Anti-hypertensive treatment ☐ No ☐ Yes

if YES → **4.2.1** *(Most recent. Select all that apply)* ☐ ACE inhibitor ☐ Ca^{2+}channel blocker ☐ ARB ☐ Thiazides ☐ Beta blocker ☐ Other

Section 6. Diabetes Related Foot Problems

	Last 12 months No	Last 12 months Yes	Previous No	Previous Yes
6.1 Peripheral neuropathy	☐	☐	☐	☐
6.2 Foot ulceration	☐	☐	☐	☐
6.3 Peripheral vascular disease	☐	☐	☐	☐
6.4 Lower limb amputation	☐	☐	☐	☐

if YES (Select all that apply) → **6.4.1** ☐ Minor ☐ Major **6.4.2** ☐ Minor ☐ Major

Section 8. Complications/Events/Comorbidities

	Last 12 months No	Last 12 months Yes	Previous No	Previous Yes
8.1 Cerebral stroke	☐	☐	☐	☐
8.2 Myocardial infarction	☐	☐	☐	☐
8.3 CABG/Angioplasty	☐	☐	☐	☐
8.4 Congestive cardiac failure	☐	☐	☐	☐
8.5 End stage kidney disease	☐	☐	☐	☐
8.6 Blindness	☐	☐	☐	☐
8.7 Sexual dysfunction	☐	☐	☐	☐
8.8 Dementia	☐	☐	☐	☐
8.9 Diabetic ketoacidosis (DKA)	☐	☐	☐	☐
8.10 Hyperosmolar hyperglycaemic state (HHS)	☐	☐	☐	☐
8.11 Severe hypoglycaemia	☐	☐	☐	☐

if YES (last 12 months) → **8.11.1 No. of episodes** ☐ 1-2 ☐ 3-5 ☐ >5

8.12 Malignancy (exclude non-melanotic skin cancers) ☐ Metastatic solid tumour ☐ Leukaemia ☐ Other ☐ Non-metastatic solid tumour ☐ Lymphoma ☐ Not applicable

8.13 Liver disease ☐ Mild ☐ Moderate/Severe ☐ Not applicable

Section 9. Renal Function & Blood Glucose Control *(Most recent in last 12 months)*

9.1 Urinary protein/albumin collected ☐ No ☐ Yes

if YES → **9.1.1 Result** ☐☐☐☐ . ☐☐ ☐ mg/L ☐ µg/min ☐ mg/24 hr ☐ ratio

9.2 Serum creatinine ☐☐☐☐ µmol/L OR ☐ Not available

9.3 HbA1c Result (%) ☐☐ . ☐ % OR ☐ Not available

9.4 HbA1c Result (mmol/mol) ☐☐☐ mmol/mol OR ☐ Not available

SAMPLE
